# Random Forest-Based Recognition of Isolated Sign Language Subwords Using Data from Accelerometers and Surface Electromyographic Sensors

**DOI:** 10.3390/s16010100

**Published:** 2016-01-14

**Authors:** Ruiliang Su, Xiang Chen, Shuai Cao, Xu Zhang

**Affiliations:** Department of Electronic Science and Technology, University of Science and Technology of China, Hefei 230027, China; srl0724@mail.ustc.edu.cn (R.S.); caoshuai@mail.ustc.edu.cn (S.C.); xuzhang90@ustc.edu.cn (X.Z.)

**Keywords:** sign language recognition, surface electromyography, accelerometer, random forest

## Abstract

Sign language recognition (SLR) has been widely used for communication amongst the hearing-impaired and non-verbal community. This paper proposes an accurate and robust SLR framework using an improved decision tree as the base classifier of random forests. This framework was used to recognize Chinese sign language subwords using recordings from a pair of portable devices worn on both arms consisting of accelerometers (ACC) and surface electromyography (sEMG) sensors. The experimental results demonstrated the validity of the proposed random forest-based method for recognition of Chinese sign language (CSL) subwords. With the proposed method, 98.25% average accuracy was obtained for the classification of a list of 121 frequently used CSL subwords. Moreover, the random forests method demonstrated a superior performance in resisting the impact of bad training samples. When the proportion of bad samples in the training set reached 50%, the recognition error rate of the random forest-based method was only 10.67%, while that of a single decision tree adopted in our previous work was almost 27.5%. Our study offers a practical way of realizing a robust and wearable EMG-ACC-based SLR systems.

## 1. Introduction

Sign language is the most natural and expressive way for communication in the hearing-impaired and non-verbal community, where information is majorly conveyed through gestures [1,2]. Sign language recognition (SLR) technology, which uses the data collected from video [3], glove [4], depth sensors [5], surface electromyography (sEMG) or accelerometers (ACC) [6] to explain gestures or signs, offers an approach to establish sign language in speech/text systems and facilitate communication in the sign language community. SLR also plays a key role in developing gesture-based human computer interaction (HCI) systems [2,7,8].

In recent works, various methods have been employed to improve the performance of SLR, which can mainly be grouped into two classes, vision-based or glove-based recognition. In video-based sign recognition, Ding *et al.* proposed a component-based recognition method to identify 38 American signs [2], and Grobel *et al.* achieved recognition of 262 isolated signs [7]. With the popularity of Kinect in the field in HCI, depth sensors play an important role in sign recognition. For instance, Paul *et al.* proposed a method for hand shape and 3D pose estimation based on depth data [5]. In glove-based recognition, Fels *et al.* realized a real-time classification system for 203 American signs [8], and Gao and his colleagues achieved high accuracy in the recognition of Chinese signs ranging from 2435 to 5177 [4,9,10,11]. However these two conventional methods both have their own practical drawbacks. Glove-based technology uses a cumbersome data glove to collect data, which can hinder the convenience and naturalness of HCI, and the vision-based technology is sensitive to environmental factors, thus these conditions limit their extensive application [6].

Differing from the approaches mentioned above, ACC and sEMG sensors provide two potential technologies for gesture capture [12]. Compared with glove-based technology, sEMG and ACC sensors are easy to wear by fixing them in a wrist strap, and compared with video-based technology, sEMG and ACC sensors can describe movement information and be less sensitive to environmental factors. sEMG sensors offer a noninvasive way of detecting muscle activity. When recorded with multiple channels, sEMG data can be used to identify various functional movements of the human body [13]. ACC sensors are widely used to sense the orientation and dynamic movements suitable for capturing large-scale motions in terms of its posture and moving trajectory. Both sEMG and ACC sensors are low-cost and portable compared with glove and visual sensors. Therefore, a much more portable SLR system could be realized via sEMG and ACC sensors. On the other hand, previous research has investigated the complementation of measurements based on sEMG and ACC sensors. The information fusion of sEMG and ACC sensors has been demonstrated to be valid in enhancing the performance of sign and gesture recognition. For example, Kim *et al.* used Support Vector Machine (SVM) and K-Nearest Neighbor (KNN) classifiers to realize recognition of seven isolated German sign language words and explored their complementary functionality [14]. Kosmidou and Hadjileontiadis successfully recognized 60 isolated Greek sign language words by applying intrinsic mode entropy on sEMG and ACC data of the dominant hand [15]. Wu *et al.* achieved a real-time American SLR system with wrist-worn motion and sEMG sensors [16]. Lin *et al.* proposed a robust gesture recognition algorithm in 10 gestures based on data of eight sEMG sensors [17]. 

In our pilot study, the combination of accelerometers and sEMG sensors was examined to synchronously detect hand movement information for the recognition of 23 hand gestures [18]. Then, a Hidden Markov Model (HMM) based decision tree was proposed as the main recognition framework for successfully recognizing 121 Chinese sign language gestures with an overall average recognition accuracy of 95.78% [6]. However, there still remains a series of problems in ACC and EMG combined SLR. The recognition accuracy of some subwords with similar features can’t be ensured. On the other hand, the HMM-based decision tree had insufficient robustness, and the recognition performance of the SLR system dropped sharply when bad samples were mixed in the training set. These problems also exist in other gesture recognition systems based on single or multi-level decision classifiers.

As a follow-up study, the purpose of this paper is to explore a random forest-based CSL subwords recognition framework. Random forest, which was first introduced by Breiman in 2001 [19,20,21], is a combination classifier, and has been proved to be effective in the field of pattern recognition [22,23]. The base classifier of random forest is a decision tree, and the idea of random forest is to build an ensemble of decision trees for improving the recognition accuracy significantly. A bagging method is applied usually to generate the training set of decision trees, which is called bootstrap sampling, to ensure the differences across different decision trees. Compared with other algorithms, random forest has been declared more accurate, and has the capability to handle bad data effectively [20]. To verify the effectiveness of random forest-based sign recognition, this study was conducted on a vocabulary composed of 121 Chinese SL subwords, which is the same one used in our previous work [1] for the convenience of comparison.

## 2. Method

**Figure 1:**
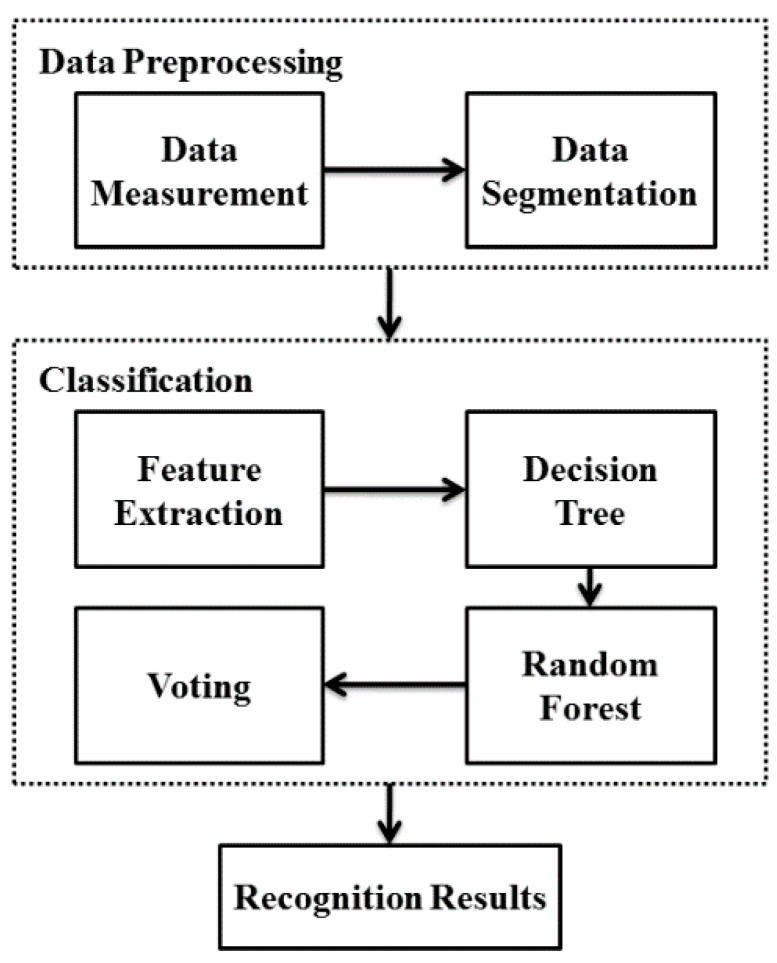
Structure of CSL subwords recognition framework based on random forest.

In this work, CSL subwords recognition was performed on the data recorded from sEMG sensors and accelerometers. The proposed framework based on random forest classification is illustrated in Figure 1. Original data is gathered by sEMG sensors and accelerometers, and a segmentation process is conducted to obtain the signal during the execution of subword from continuous data stream. Then features are extracted based on the rules of decision tree, and finally random forest is generated and adopted as the classifier. All data processing was done using MATLAB R2013a (The Mathworks, Inc., Natick, MA, USA).

### 2.1. Data Measurement

**Figure 2:**
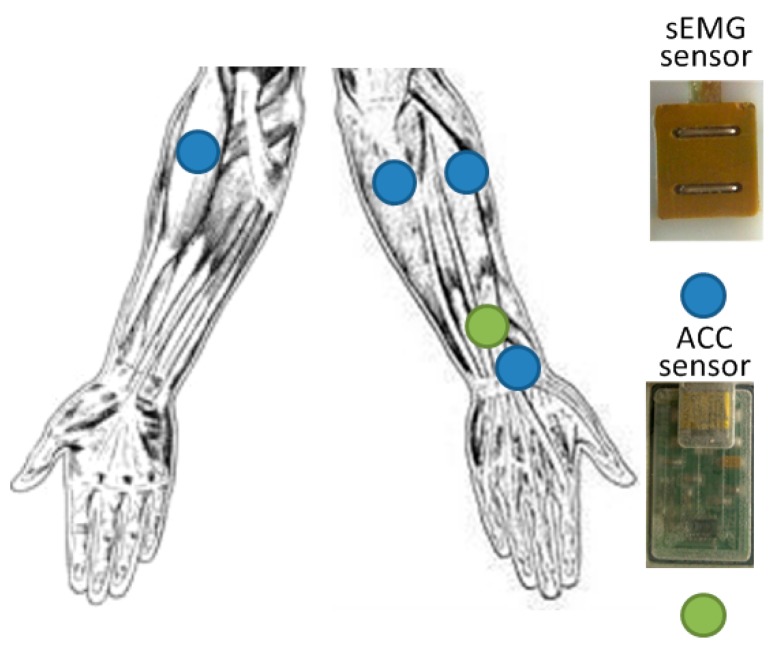
Positions of sEMG and ACC sensors on the right arms.

A self-made acquisition device was employed to acquire the data of 121 CSL subwords. Eight self-made sEMG and two MMA7361 (Freescale Semiconductor, Inc., Austin, TX, USA) 3-axis ACC sensors were placed symmetrically around the left and right forearms [6]. Figure 2 illustrates the concrete placement plan in our study. For each hand, the 3-axis ACC and one sEMG sensor were placed near the back of the wrist and other three sEMG sensors were located on the forearm close to elbow. The sEMG sensor near the wrist was used to detect the activity of the extensor minimi digiti, and the other three sEMG sensors near the elbow were used to detect the activity of the palmaris longus, extensor carpi ulnaris and extensor carpi radialis muscles. The schemes of the positions of sEMG sensors have been proven efficient and feasible to detect muscle activity from finger and wrist movements. [24] Near the wrist, a round electrode (Dermatrode; American Imex, Irvine, CA, USA) was placed with a wristband on the ulnar side of the right forearm as the reference electrode. 

The raw signal of each sensor was amplified by AD8220 and AD8698 (Analog Devices, Inc., Norwood, MA, USA) with a 20–1000 Hz band pass filter, and then signals were digitized by a data acquisition card (NI PCI-6036E; National Instruments, Austin, TX, USA) at 1 kHz sampling rate. The data of 121 CSL subwords is the same as that in our previous work [1].

### 2.2. Data Segmentation

The objective of this step is to extract subwords from CSL sentences, and to remove the movement epenthesis between two subwords. The main work of data segmentation is to determine the start and end points of muscle activity. Because the dominant hand participates in all the 121 CSL subwords, sEMG signals from the dominant hand were used for data segmentation [25,26], and the proposed data segmentation approach consists of four steps described as follows:

1. Step 1: To divide the four-channel sEMG signals into time-shifting chunks with a sliding window. With an increased length of sliding window, both the computation complexity and the accuracy will decrease. In this study, the sampling rate of the sEMG sensor is 1 kHz and the duration of a CSL subword is generally about one second. Based on segmentation experiments with different window size and overlap, 128 ms (128 samples) window length and 64 ms (64 samples) window increment was confirmed to be able to balance the computational complexity and segmentation accuracy.
2. Step 2: To calculate the average energy of four sEMG channels for each windowed data chunk according to Equation (1), where *x* means the signal, *t* means the index of the data chunk and *i* means the index of the sEMG channel:
  (1) $$E_{w}(t)=\frac{1}{4}{\sum_{i=1}^{4}\frac{1}{N}\sum_{n=0}^{N-1}\left|x_{i}\left[n\right]\right|}^{2}$$
3. Step 3: To detect the starting index t_s_ and the ending index t_e_ of each subword segment. In this step, the time series {E_W_(t)} were compared with a predefined threshold T_R_, which was set as the twenty percent of the mean of the E_W_(t) of the signer’s maximal voluntary contraction. When E_W_(t), E_W_(t + 1) and E_W_(t + 2) are larger than T_R_ but E_W_(t − 1) and E_W_(t − 2) are lower than T_R_, the starting point (t_s_) is determined. Similarly, when E_W_(t), E_W_(t − 1) and E_W_(t − 2) are larger than T_R_, but E_W_(t + 1) and E_W_(t + 2) are lower than T_R_, the end point (t_e_) is found. t_e_ − t_s_ + 1 should be larger than 8 to avoid the influence of instant noise.
4. Step 4: To enforce the same boundaries on the sEMG channels of the non-dominant hand and the ACC channels of both hands based on the boundaries detected in Step 3.

**Figure 3:**
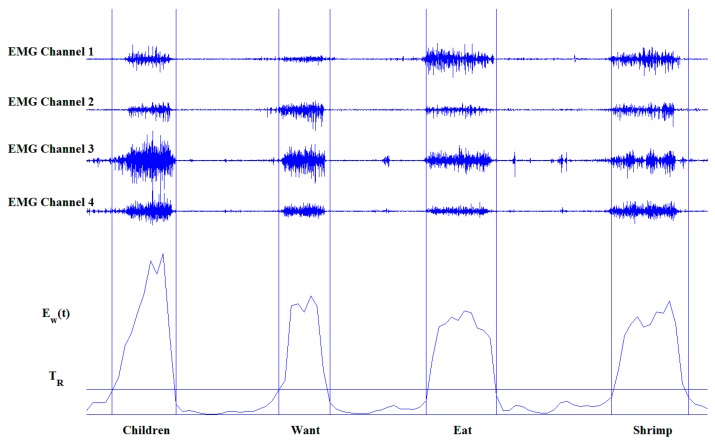
An example of the segmentation of a CSL sentence “Children want to eat shrimp,” which is constituted by four CSL subwords “children”, “want”, “eat”, “shrimp”.The predefined threshold T_R_ is set as horizontal line. And the vertical lines indicate the subword boundaries.

Several other methods also were proposed to detect the boundaries of the muscle activity segments beside the average energy used in our study, such as amplitude envelop and sample entropy. The average energy was chosen due to its ease of implementation and high practicality. Figure 3 shows an example of the segmentation of a CSL sentences.

### 2.3. Feature Extraction

Feature extraction attempts to efficiently describe the original subword segment using a set of parameters that can ensure the optimal performance of the classifier [27]. In our method, features are selected to meet the requirements of the base classifier in random forest. And the details of the adopted features are described as follows:

1. Mean Absolute Value (MAV) [27]: Describes the energy of signals. The MAV of sEMG or ACC signal of non-dominant hand is used to distinguish one-handed or two-handed subwords:
  (2) $$MAV=\frac{1}{N}\sum_{n=1}^{N}\left|x(n)\right|$$
  where *x*(n) represents the sEMG or ACC signal in a segment and *N* denotes the length of the segment.
2. Autoregressive (AR) Coefficient [24]: AR model describes the current signal *x*(n) as the linear combination of previous *k* samples *x*(*n* − *k*) plus white noise *w*(n). AR coefficients (*a_k_*) have been proven to be effective in SLR. The definition of the AR model is given by Equation (3), and *p* is the order of the AR model:
  (3) $$x(n)=w(n)-\sum_{k=1}^{p}a_{k}x(n-k)$$
3. Mean: Describes the amplitude level of signals. The mean of the acceleration signal is used to describe the orientation of each subword:
  (4) $$Mean=\frac{1}{N}\sum_{n=1}^{N}x(n)$$
  where *x*(n) represents the ACC signals in a segment and N denotes the length of the acceleration signal.
4. Variance (VAR) [27]: Describes the intensity of the signal changing with time. The VAR of the acceleration signal is used to judge the movement range of a subword:
  (5) $$Var=\frac{1}{N-1}\sum_{n=1}^{N}\left(x(n\right)-Mean{)}^{2}$$
5. Linear Predication Coefficient (LPC): LPC model describes each sample of signals as a linear combination of previous *k* samples *x*(n − k) as Equation (6) shows
  (6) $$x(n)=-\sum_{k=1}^{l}l_{k}x(n-k)$$
  where *p* is the order of LPC and *l*_k_ denotes the *k*-order coefficient.

### 2.4. Random Forest-Based Classification

In this study, random forest was proposed as the main classification framework. Random forest is a combined classifier consisting of many tree-structured classifiers. In the proposed method, an improved decision tree, which can fuse features extracted from sEMG and ACC signals of subword, is adopted as the base tree-structured classifier of the random forest. All improved decision trees in random forest have the same structure, and a random vector is used to generate a bootstrap sample as the training set of each improved decision tree to ensure the difference of parameters of decision trees. The random forest grows an ensemble of trees and lets them vote for the most popular class [22,23].

**Figure 4:**
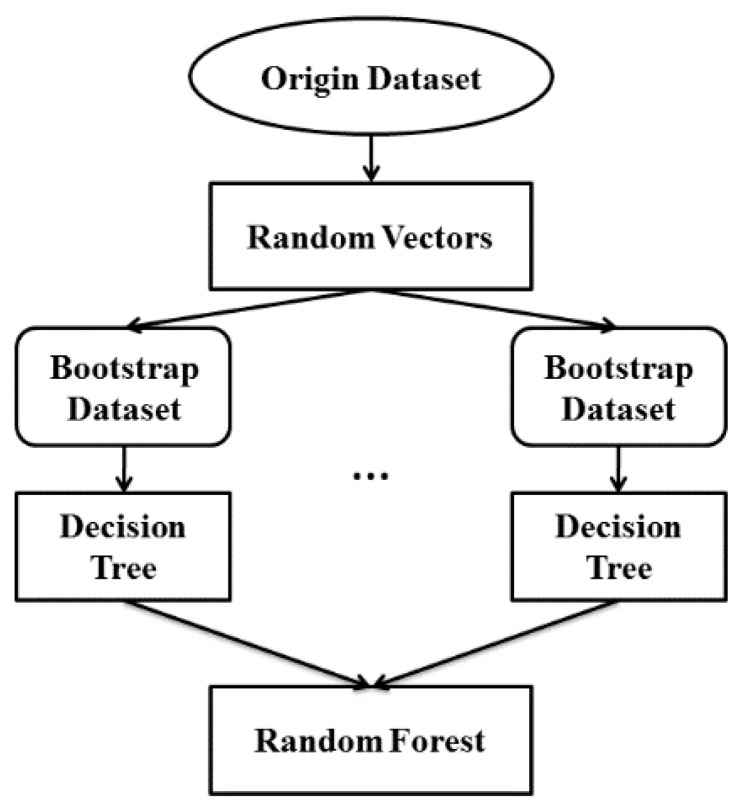
Structure of proposed random forest algorithm. The structure of the decision tree is detailed in Figure 5.

The structure of the proposed random forest algorithm is shown in Figure 4. Given the original training set with $N=\sum_{i=0}^{121}N_{i}$ samples is composed of 121 CSL subwords (the *i*-th subword has $N_{i}$ samples), a random vector $v=\{v_{1},v_{2},...v_{i},...,v_{121}\}$ ($v_{i}$ is the random vector with $N_{i}$ elements of subword *i*, and each element in the random vector $v_{i}$ is a random value in the range of 1 to $N_{i}$, representing the sample index.) is generated to extract samples in the original training set at random with replacement to build the training set of a decision tree in the random forest. The resulting set was formed as the training set of the decision tree with *N* samples, called the bootstrap sample. Then the parameters of each sub-classifier of the decision tree were trained according to the pre-set rules. Due to the differences of the training sets, the parameters of the decision tree varied. This process to build the decision tree continued until the scale of random forest met the requirements that the stability of the recognition rate was ensured. In the testing phase, the forest collects the results of each decision tree, and set the class with the maximum number as the final result.

**Figure 5:**
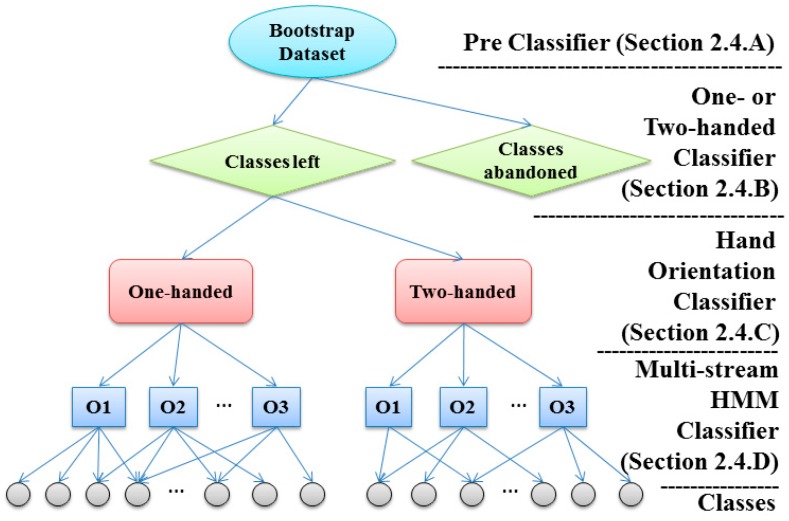
Structure of the improved decision tree in random forest.

In order to ensure the practical performance of the proposed method, the base classifier of the proposed random forest algorithm consisted of a pre classifier, one- or two-handed classifier, hand orientation classifier and a multi-stream HMM classifier. Figure 5 illustrates the structure of the improved decision tree. And the details of individual steps were described in the following.

#### 2.4.1. Improved Decision Tree of Random Forest

A decision tree is the base classifier of random forest, the result of which is collected by random forest to vote for the most popular class [22,23]. The hierarchical tree structure consists of root nodes, internal nodes and leaf nodes based on a series of rules about the attributes of classes in non-leaf nodes, where each leaf node denote a class [4]. The origin input samples were first fed into the root node of the decision tree. When the samples reached a no-leaf node, they were diverted down different branches based on rules associated with the corresponding node. Finally, the samples of the same subword were gathered to the leaf node. In our previous research work [6], the decision tree was employed as the main framework and proven to be able to fuse various information sources and reduce the computational complexity.

The decision tree in our previous study was constructed by a one- or two-handed classifier, a hand orientation classifier, and a multi-stream HMM classifier. As shown in Figure 5, each non-leaf node denotes a classifier associating with the corresponding subword candidates. It was found that subwords with similar features are prone to misclassification, which results in compromised recognition accuracy. To avoid this situation, similar subwords need to be separated prior to being fed to the leaf node (the HMM classifier) at the final level of the decision tree. In this study, a sub-classifier which we called the pre-classifier is added to separate similar subwords at the top layer of the improved decision tree. In the testing phase, the unknown input data are first fed into the pre-classifier, then into the one- or two-hand classifier and the hand orientation classifier, and then finally into the multi-stream HMM classifier to get the final result.

##### A. Pre-Classifier

**Figure 6:**
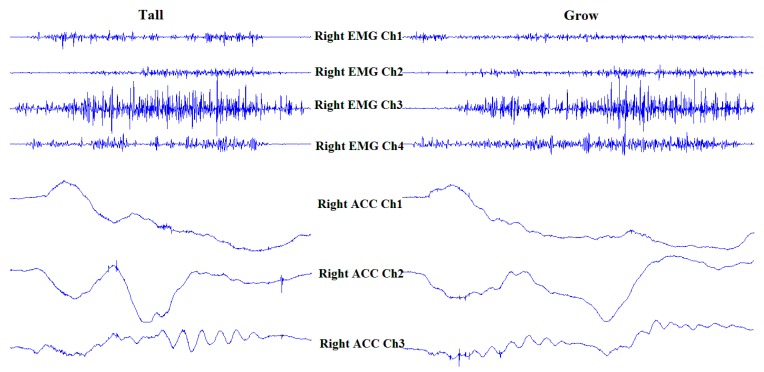
SEMG and ACC signals of subwords “tall” and “grow”.

As mentioned above, the purpose of the pre-classifier is to distinguish some subword pairs. A subword pair was defined as two easily confused subwords which involved substantially similar gestures. The subwords “tall” and “grow” as shown as an example. The sign for the subword “tall” involves one hand held straight with the palm down, while moving from the chest to the top of the head. Similarly, the sign of the subword “grow” involves one hand held straight, palm down, while slowly moving up in the front of the chest. As Figure 6 shows, the sEMG and ACC signals of the subword “tall” and the subword “grow” are very similar. Due the similarity of action of the subword pair, it is necessary to separate the subword pair into different branches of the decision tree.

Based on the analysis of the execution of the sign language subwords, 154 easily confused subword pairs (involving 88 subwords) involving similar definitions and implementation processes were selected from the 121 CSL subwords to construct the pre-classifier. A support vector machine (SVM) was employed to separate two subwords in one pair using the MAV, VAR and fourth-order LPC of each channel of EMG and ACC signal. Pre-classification was based on data from the dominant hand. Since there are four EMG channels and a 3-D accelerometer in each hand, the input vector of SVM is 7 × 6 = 42 dimensions. In the training phase, data samples from two similar subwords were selected to train a SVM classifier. Therefore, the pre-classifier is made up of a set of SVM classifiers. In the testing phase, given the combination of features of a subword segment, the output of each SVM classifier represents a potential final result, and the other class of SVM denotes an unlikely final result. The output of the pre-classifier is the original set of 121 subwords with the unlikely results removed.

After the pre-classifier, the node was split into two child nodes, one child node denotes the remaining set of potential candidates called “classes left”, and the other denotes the unlikely candidate set called “classes abandoned” which never appear in the following layer in the decision tree. As the top layer of the improved decision tree, the pre-classifier plays a role in ruling out impossible subwords, so the output of the pre-classifier is the remaining set of possible subwords. 

##### B. One- or Two-Handed Classifier

The one- or two-handed classifier was employed to determine whether the muscle of the non-dominant hand is active during the execution of a subword [6]. For one-handed subwords, the non-dominant hand stays motionless during the subword execution, so the MAV of the sEMG signals and the mean and standard deviations of the ACC signals measured from the non-dominant forearm are significantly lower than those of two-handed subword. The difference can be easily distinguished by SVM, which has been widely used in distinguishing two kinds of samples of different categories [14].

Data samples of the training set were marked as one-handed or two-handed, and then fed into the SVM (the input vector is 10 dimensions) to train the maximum-margin hyper-plane. The decision result of the classifier depends on which side of hyper-plane the sample test data vector lies in.

As the next layer in the decision tree, the one- or two-handed classifier handles the output candidate set from the pre-classifier, and attempts to reduce the size of the candidate set further. Therefore, if the subword segment is judged as one-handed, the two-handed classes will no longer be included in the candidate. 

##### C. Hand Orientation Classifier

**Figure 7:**
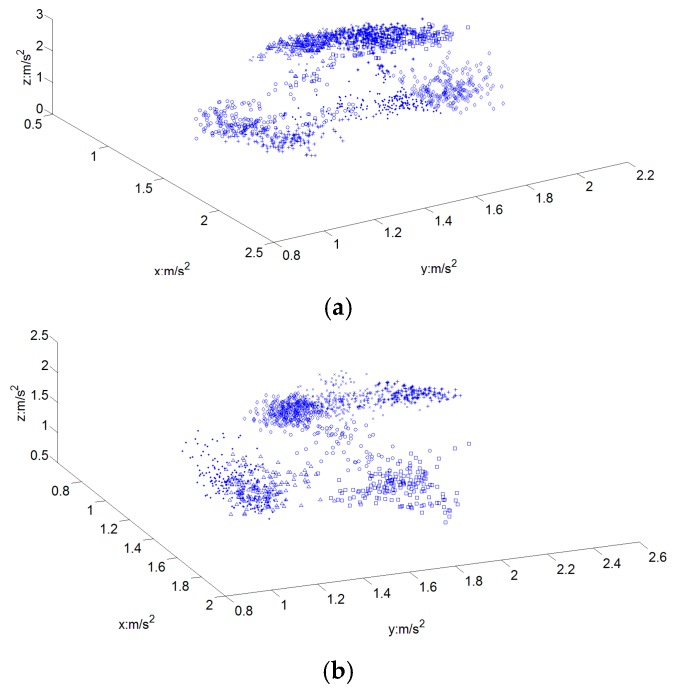
Distributions of the ACC mean values of dominant hand. (**a**) Distribution of one-handed subwords; (**b**) Distribution of two-handed subwords.

The target of this layer of the improved decision tree is to decide the orientation of the subword. The description of hand orientation usually includes two terms, one is the direction to which the hand is pointing, and the other is the direction of the palm [6]. The value of the 3-axis ACC signal is different in different hand orientations due to the projection of gravity. Therefore, the mean value of each of the three ACC signals can effectively reflect the information of hand orientation [6]. Figure 7 shows the distribution of the ACC means of the dominant hand of one signer, composed of 1542 samples of 59 one-handed subword candidates and 1280 samples of 62 two-handed subword candidates. As shown in Figure 7, there are 7~8 kinds of possible hand orientations in the 121 CSL subwords.

Before the hand orientation classifier, the subword candidate set had been split into two branches: one-handed and two-handed subwords. Thus the classifier was trained independently for two branches. In the training phase, one-handed subword segments and two-handed subword segments were collected separately and assigned to different cluster centers which denote the pattern branch of hand orientation by fuzzy K-means algorithm. After this process, a Linear Discriminant Classifier (LDC) was employed to determine which hand orientation group the input data belongs to. Using the Euclidean distance as the judgment standard, the result is the pattern branch which has the minimum distance to the input sample. For both one-handed and two-handed subwords, the K parameter of fuzzy K-means was set as 8 in the hand orientation classifier according to the distributions of the ACC mean values of the dominant hand.

##### D. Multi-Stream HMM Classifier

Hidden Markov Model (HMM) is a powerful tool to model sequential data. In our previous work, Multi-stream HMM was used successfully to model the sign language subwords [1]. In order to build the multi-stream HMM, the sEMG and ACC signals in a subword segment need to be converted into a set of feature sequences using a feature extraction process. For each hand, the 3-axis ACC signals were linearly down-sampled to 64-point time sequences to normalize the movement duration, and each consecutive sEMG signals were separated into frames using the sliding windows method with a frame size of 128 and a step size of 64. The MAV and 4th-order linear prediction coefficients of each channel were then calculated and concatenated as a 20-D feature vector for each frame. Therefore, a set of two-stream observations, *O* = {*O_A_*,*O_E_*}, were formed for each subword segment, where *O_A_* denotes a sequence of feature vectors from ACC, and *O_E_* denotes a sequence of feature vectors from sEMG. The features of one frame represent a point in the observation feature space as described in the HMM model.

One-handed subwords and two-handed subwords were modeled separately. For one-handed subwords, only the ACC and sEMG feature vector sequences of the dominant hand were used to train a multi-stream HMM denoted as *R_C_* = {*R_CA_*,*R_CE_*}, where *C* is the index of the subword candidate. Two continuous HMMs were utilized to model each stream of $O_{A}^{R}$ and $O_{E}^{R}$ as *R_CA_* and *R_CE_, * respectively, and each model for a single stream was trained using the Baum-Welch algorithm. The structure of a HMM model consists of five states and three mixture components. For two-handed subwords, two separate multi-stream HMMs were trained to model the two hands, using the observations of both hands denoted as *O^R^* and *O^L^*. The training of multi-stream HMM for the non-dominant hand *L_C_* was carried out by the same procedure as *R_C_*.

In the testing phase, the pattern branch judgement starts with the candidate set from the hand orientation classifier and then feeds the unknown sample into the multi-stream HMM classifier. For one-handed subwords, the likelihood *P_C_* was calculated as Equation (7) using the forward algorithm to determine the likelihood of the observation of the input sample. The recognition result is the class whose multi-stream HMM for the dominant hand achieves the highest likelihood.

(7) $$P_{C}=\delta_{A}P(O_{A}^{R}|R_{CA})+\delta_{E}P(O_{E}^{R}|R_{CE}),c^{*}=\arg\max P_{C}$$

where 1 ≤ *c* ≤ *C*, *δ_A_* and *δ_E_* denote the weight of ACC and sEMG respectively, *R_CA_* and *R_CE_* denote the ACC and sEMG part of the multi-stream HMM *R_C_*, $O_{A}^{R}$ and $O_{E}^{R}$ denote the ACC and sEMG part of the observation of right hand *O^R^*, and c* is the final result of the test sample.

For two-handed subwords, the likelihoods of dominant and nondominant hand were calculated separately, and the recognition result of the decision tree was the class whose multi-stream HMMs for both hands achieved the highest combined likelihood:

(8) $$c^{*}=\arg\max(P(O^{R}|R_{C})+P(O^{L}|L_{C}))$$

#### 2.4.2. Random Forests

Generating different decision trees are necessary to build the random forest . Assuming that the size of the training set corresponding to each word is {*N_1_*,*N_2_*,…,*N_i_*,..,*N_121_*}, where *i* denotes the index of subwords, and the total number of the training set is *N*, the random forest was grown as follows:

1. Generate the training set of each decision tree in random forest. For the subword *i*, extract sample with *N_i_* times of this subword in the origin training set at random with replacement. The final result will be the training set of the decision tree used to train the parameter of the decision tree by the rules previously described.
2. Determine whether the number of decision trees meet the preset scale of random forest, if not, repeat steps above. The preset scale was determined by calculating the recognition rate under different scales of random forest. The smallest scale which best ensured accuracy greater than 90% was selected. In the testing phase, each decision tree in the random forest generated a recognition result, and the final result of the random forest was considered to be the most frequent class in the recognition results of all decision trees.

## 3. Results and Analysis

### 3.1. Subwords Recognition Results

In the data collection experiments, five right-handed subjects were recruited with approval and consents of the subjects as signers (labeled as S1–S5), three male and two female, aged between 20 and 26 years old. One signer was a CSL teacher and the others used to do some volunteer work for hearing-impaired children in a local school for people with disabilities. Both groups of signers were all healthy with no history of joint and neuromuscular diseases and performed CSL proficiently in the experiment. 200 representative CSL sentences constituted by a vocabulary of 121 subwords were selected as the set of subwords used for the experiments, and each sentence was executed three times. Detailed information is available in [1]. Because there are three repetitions of each CSL sentence, the recognition of subwords was carried out using a three-fold cross-validation scheme where two of the repetitions were used as the training set and the remaining repetition used as the testing set.

#### 3.1.1. Recognition Results of the Improved Decision Tree

To verify the recognition performance of our improved decision tree with pre-classifier (IDT), the recognition accuracy after each sub-classifier of the tree was calculated under the training and testing set mentioned above, and compared with the results of the decision tree without pre-classifier (DT) in our previous work. Table 1 shows the results of each signer. The results of the hand number and hand orientation classifier were obtained without pre-classifier, because the pre-classifier makes a little influence on these two sub-classifiers.

**Table 1:** Recognition results of sub-classifiers of the improved decision tree and decision tree.

		S1	S2	S3	S4	S5	Mean ± Standard Deviation
One- or Two-handed		99.79%	99.55%	99.40%	100%	99.51%	99.65% ± 0.24%
Hand Orientation		98.89%	97.67%	98.74%	98.99%	98.07%	98.47% ± 0.57%
Multi-stream HMM	IDT	98.63%	97.36%	98.31%	98.36%	97.96%	98.12% ± 0.49%
	DT	95.79%	94.78%	97.32%	97.04%	95.15%	96.02% ± 1.13%

As shown in Table 1, it is clear that the improved decision tree with pre-classifier proposed in this study outperformed the decision tree without pre-classifier. Due to the addition of the pre-classifier, the improved decision tree strips out some subwords from the candidate set before the multi-stream HMM classifier, improving the recognition accuracy.

#### 3.1.2. The Influence of the Scale of Random Forest on Recognition Results

**Figure 8:**
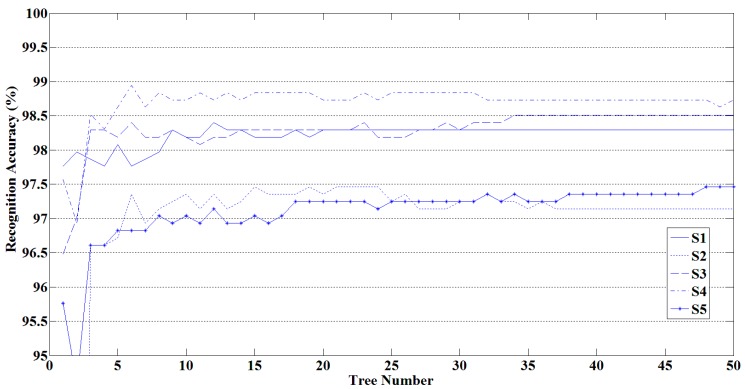
Relationship between the number of decision trees in a random forest and the recognition accuracy.

The recognition performance is related to the scale of the random forest due to the rule of voting. To determine the suitable scale of random forest in the process of classification, a random forest-based recognition experiment with different number of decision trees was conducted. Figure 8 shows the relationship between the number of decision trees in random forest and the recognition accuracies of five signers. From Figure 8, we can observe that the recognition accuracy gradually increases and stabilizes with the increase of the number of decision trees. When the random forest contains more than 30 decision trees, the recognition result of random forest remains at a high level.

#### 3.1.3. Recognition Results of Random Forest-Based Method

**Figure 9:**
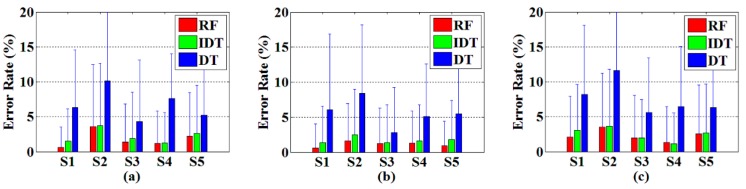
Subword recognition results achieved by the proposed RF method in comparison to the IDT and DT for five different signers (S1–S5) using three-fold cross validation. (**a**) the first repetition dataset for testing; (**b**) the second repetition dataset for testing; (**c**) the third repetition dataset for testing.

The subwords classification error rates achieved by the proposed method (denoted as RF, the random forest contains 30 decision trees) in comparison to the results of the single decision tree method (used in our previous [6] and denoted as DT) and single improved decision tree method (denoted as IDT) for five signers are shown in Figure 9. The classification error rates of the proposed RF method are in the range of 0.63% to 3.61%, while those of DT ranges from 2.83% to 11.60% and IDT ranges from 1.15% to 3.74%. It is clear that the proposed random forest method and the improved decision tree method consistently outperformed our previous method.

### 3.2. Robustness Testing Results

**Figure 10:**
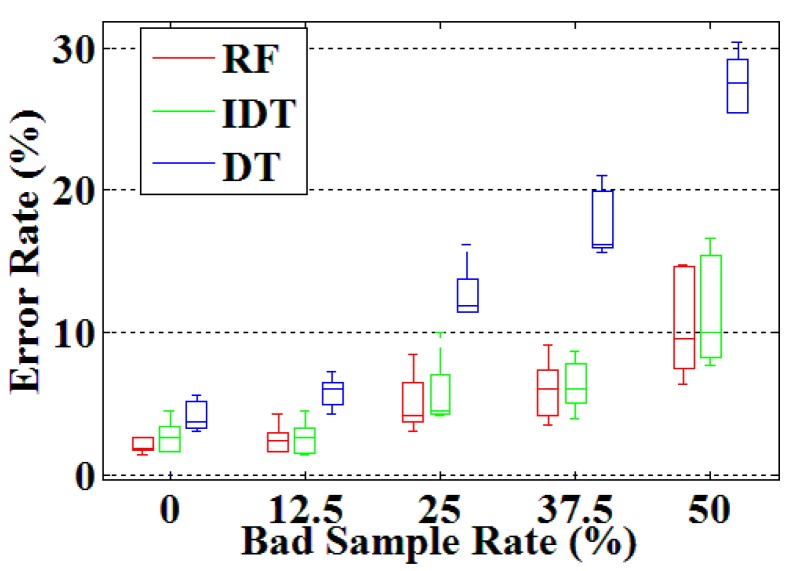
Results of robustness testing experiment. The horizontal axis denotes the proportion of bad samples in training set and the vertical axis denotes the error recognition rates of 121 CSL subwords.

An experiment was conducted to verify the robustness of the random forest-based method in gesture recognition. After analyzing the data set of 200 CSL sentences, it was found that each of 121 CSL subwords at least appeared four times. To ensure the same proportion of bad samples, we picked the first four samples of each subword in each repetition of 200 CSL sentences to form the dataset of robustness testing experiment, so each dataset contained 12 samples for each subword. For a given subword, the robustness testing experiments were executed by replacing the samples in the training set with the samples of a similar subword according to the easily confused pair selected in the pre-classifier. The number of bad samples was in the range of 0 to 4, and we randomly selected samples in the training set to replace. As mentioned previously, the proportion of the training set to testing set in the subword recognition was 2:1. The first and the third groups were used as training set and the second group as testing set. We randomly replaced a number of subword samples in increasing increments with created training sets with an incorrect sample rate ranging from 0 to 50%. Figure 10 shows the results of robustness testing experiment achieved by the proposed method (denoted as RF) in comparison to the improved decision tree method (denoted as IDT) and single decision tree method (denoted as DT) for five signers.

As shown in Figure 10, random forest and improved decision tree adopted in our study performed better in the aspect of robustness. When the proportion of bad samples in the training set reached 50%, the recognition error rates of random forest and improved decision tree are less than 15%, while that of decision tree adopted in our previous work is larger than 25% in this condition. The *p*-value of RF and IDT is 0.36, and RF and DT is 1.22 × 10^−5^ (Because there is no obvious evidence to show that the results obey the normal distribution, Wilcoxon Sign Rank Test was adopted.). So we draw a conclusion that RF outperformed DT in the aspect of robustness. Compared to IDT, the random forest doesn’t show any obvious advantages in robustness, but in the aspect of recognition accuracy, RF has a certain advantage.

## 4. Discussion and Future Work

This study confirmed our previous finding that the combination of the sEMG and ACC signals is a promising sensing technique for the implementation of a practical SLR system. In our proposed random forest-based method for the recognition of Chinese Sign Language (CSL) subwords, the use of an improved decision tree increases the likelihood of each decision tree in random forests to get a correct result. The application of bagging method also ensures that each decision tree is trained by a different bootstrap sample dataset, which is the foundation of its robustness, while the voting principle helps to avoid the interference of wrong samples. Based on the recognition results of 121 frequently used CSL subwords, the superior performance of the random forests method in the aspect of precision and robustness was verified. Compared with related studies, this study achieved relatively satisfactory results with 98.25% recognition accuracy.

One main limitation of this work is long time required to build the random forest. Since a forest is composed of many decision trees with the same structure, the time required to build the random forest classifier is much longer than the time to build a decision tree. Moreover, in the voting phase of the proposed method, each decision tree was set to the same weight, and which was a compromise made due to the complexity of the random forest in SLR. Additionally, the random selection strategy of features of random forest was not applied in the proposed method, which caused each decision tree in the random forest to have the same structure. There are also some limitations in actual application; the method was proposed for a user-specific SLR system. User fatigue and changes in sensor location may influence the long-term performance of the system.

Based on these limitations, the application of random forest in a real SLR system still requires more work in the future. Optimization strategies should be adopted to improve time efficiency, and the out of bag error should be considered to determine the weight of decision tree in improving the precision of recognition. Features that can ensure the robustness of the classification should be explored, and decision trees with different structures should be considered. Furthermore, our future work will also attempt to move toward user-independent recognition while considering the long-term performance in real-world applications.

## 5. Conclusions

In this paper, a random forest-based method was proposed to improve CSL subword recognition using ACC and sEMG data. In our method, a random forest algorithm was employed as the main classification framework, and an improved classifier, which added a pre-classifier to remove the easily confused candidate subwords, was designed and used as the base classifier of random forest. Recognition experiment results of 121 frequently used CSL subwords demonstrated the validity and robustness of the proposed method. As we know, ACC and sEMG sensors could enable more portable SLR systems compared with the data glove or camera used in conventional SLR systems. Our study provides a possibility for the realization of high accuracy, robustness, and wearable EMG-ACC-based SLR systems that could be used by the sign language community in daily life. Towards this ultimate goal, further work is still needed, especially to reduce the training and recognition time, which is the key to obtain a fully practical SLR system.
